# Infectious bursal disease virus: predicting viral pathotype using machine learning models focused on early changes in total blood cell counts

**DOI:** 10.1186/s13567-023-01222-5

**Published:** 2023-10-30

**Authors:** Annonciade Molinet, Céline Courtillon, Stéphanie Bougeard, Alassane Keita, Béatrice Grasland, Nicolas Eterradossi, Sébastien Soubies

**Affiliations:** 1 Agence Nationale de Sécurité Sanitaire de L’alimentation, de L’environnement Et du Travail, 41 Rue de Beaucemaine, 22440 Ploufragan, France; 2 INRAE-ENVT, UMR 1225 IHAP, 23 Chemin Des Capelles, 31076 Toulouse CEDEX 3, France

**Keywords:** Infectious bursal disease, Gumboro, pathotype, predictive model, machine learning, blood formula

## Abstract

Infectious bursal disease (IBD) is an avian viral disease caused in chickens by infectious bursal disease virus (IBDV). IBDV strains (*Avibirnavirus* genus, *Birnaviridae* family) exhibit different pathotypes, for which no molecular marker is available yet. The different pathotypes, ranging from sub-clinical to inducing immunosuppression and high mortality, are currently determined through a 10-day-long animal experiment designed to compare mortality and clinical score of the uncharacterized strain with references strains. Limits of this protocol lie within standardization and the extensive use of animal experimentation. The aim of this study was to establish a predictive model of viral pathotype based on a minimum number of early parameters measured during infection, allowing faster pathotyping of IBDV strains with improved ethics. We thus measured, at 2 and 4 days post-infection (dpi), the blood concentrations of various immune and coagulation related cells, the uricemia and the infectious viral load in the bursa of Fabricius of chicken infected under standardized conditions with a panel of viruses encompassing the different pathotypes of IBDV. Machine learning algorithms allowed establishing a predictive model of the pathotype based on early changes of the blood cell formula, whose accuracy reached 84.1%. Its accuracy to predict the attenuated and strictly immunosuppressive pathotypes was above 90%. The key parameters for this model were the blood concentrations of B cells, T cells, monocytes, granulocytes, thrombocytes and erythrocytes of infected chickens at 4 dpi. This predictive model could be a second option to traditional IBDV pathotyping that is faster, and more ethical.

**Supplementary Information:**

The online version contains supplementary material available at 10.1186/s13567-023-01222-5.

## Introduction

Infectious bursal disease (IBD), also known as Gumboro disease, is a viral disease affecting chickens (*Gallus gallus*) mostly between 3 and 7 weeks of age. This worldwide spread disease was first observed in 1957 [1] in the United States of America and its agent was characterized in 1969 [2]. IBD clinical signs are generally non-specific and comprise: diarrhea, ruffled feathers, general weakness and sometimes mortality. However, oedema and haemorrhages in the bursa of Fabricius (BF) (principal target tissue of IBDV) during the acute phase of infection can be pathognomonic of pathogenic IBD virus (IBDV). Bursal atrophy is frequent in birds surviving acute IBD and these birds are immunosuppressed [3]. The clinical picture associated with IBD depends on IBDV pathotype but can also depend on host genetics [4, 5], immunity (active or passive) [6], and possibly complicating pathogens [7]. IBDV is a non-enveloped virus with a bi-segmented double-stranded RNA genome belonging to the family *Birnaviridae*, genus *Avibirnavirus*. Two IBDV serotypes exist but only serotype 1 may cause disease.

An IBDV nomenclature was recently proposed based on the genotypic classification of both genomic segments [8, 9]. This classification distinguishes nine genogroups for segment A and five for segment B. In this nomenclature, the genetic structure of a given IBDV strain is thus represented by a genotype AxBy, with x ranging from 0 to 8 and y from 1 to 5.

Additionally, IBDV strains are classified into four pathotypes, corresponding to distinct clinical pictures [10]. First, nonpathogenic or mild strains cause neither clinical signs nor lesions or immunosuppression. Second, IBDV strains cause little or no clinical signs. Among such strains are several categories of live attenuated IBD vaccines. Currently available live IBDV vaccine strains are considered as “intermediate” strains since they do not induce clinical signs but may cause a transient immunosuppression, the latter feature being more pronounced with live vaccines designated as “invasive”, “intermediate plus” or “hot”. Then, virulent strains, that comprise, among others, classic and US antigenic variant strains, are associated with various degrees of clinical signs, lesions and immunosuppression, with infection by classic virulent viruses possibly leading to death. Finally, very virulent strains have the same clinical characteristics as the classic pathogenic strains but induce a mortality rate twice as high at least [11]. Accordingly, the distinction between the virulent and very virulent pathotypes is based on the comparison of the mortality rate and clinical picture induced by reference strains and the uncharacterized strain. Pathotyping of IBDV is currently only determined through animal experiments, the reliability of which requires standardized experimental conditions, and the use of reference viral strains of known pathotype to allow comparison between laboratories. These conditions are seldom met or increase significantly the cost of the experiments.

Given the immunosupressive nature of IBDV infection and the physiopathological differences associated with pathotypes, the host immune system/virus interplay has been investigated, however these mechanisms are not very well understood. Direct impact of IBDV on immune cells like B cells [12], macrophages [13], or dendritic cells [14] has been documented as well as differential infiltration of organs by T cells [15, 16], granulocytes [17] and macrophages [13]. Since IBDV pathotypes lead to different levels of immunosuppression, possibly reflected by early changes in immune blood cell concentration, the current study investigated the relevance of these changes as indicators of the viral pathotype. In the present study, we simultaneously evaluated and compared the in vivo viral behavior of the four IBDV pathotypes, with special attention to early changes in total blood cell counts of infected chickens and viral replication levels in the BF, in order to possibly identify, by implementing machine learning, biological indicators allowing a more straightforward determination of the different IBDV pathotypes.

## Materials and methods

### Viral strains

IBDV strains listed in Table 1 were propagated ex vivo on B cell primary cultures as described below.

**Table 1:** **Viral strains used in this study, their pathotype and genotype classification.**

Strain (Study identifier)	Viral pathotype	Genotype [9]	References
Intermediate vaccine (i vaccine)	Attenuated (Intermediate vaccine)	A1aB1	[68, 69]
Intermediate + vaccine (i + vaccine)	Attenuated (Intermediate + vaccine)	A1aB1	[69]
80 Ga (im1)	Virulent (Strictly immunosuppressive)	A4B1	[69, 70]
Variant E (im2)	Virulent (Strictly immunosuppressive)	A2B1	[71]
F52/70 (Cla)	Virulent (Classic)	A1aB1	[72, 73]
89163 (Vv1)	Very virulent	A3B2	[69, 74]
HLJ0504 (Vv2)	Very virulent	A3B3	[73]

### Propagation of IBDV on primary chicken bursal cells and preparation of viral stocks

Bursa of Fabricius (BF) were aseptically collected from four-to-ten-week-old specific-pathogen-free (SPF) White Leghorns chickens (ANSES, Ploufragan, France) and were processed as previously described [18]. Bursal cells were maintained in lymphocyte culture medium at 40 °C in a humidified 5% CO_2_ incubator. This medium was prepared using Iscove’s modified Dulbecco’s Medium (IMDM, Fisher) with L-glutamine and HEPES (reference 21980-032, Gibco, Thermo Fisher) supplemented with 8% FBS (Fetal Bovine Serum), 2% SPF chicken serum (ANSES, Ploufragan, France), 1X insulin transferrin selenium (reference 41400–045, Gibco, Thermo Fisher), 50 µM beta-mercaptoethanol, 1 µg/mL Phorbol 12-myristate 13-acetate (PMA, reference tlrl-pma, Invivogen), penicillin (200 IU/mL), streptomycin (0.2 mg/mL) and fungizone (2 µg/mL). PMA was reconstituted as previously described [18]. Bursal cells were diluted into phosphate buffered saline (PBS) 1X, pH = 7.2 (Gibco reference 20012027) containing 0.1% (m/v) erythrosin B (reference 200964, Sigma-Aldrich) and counted in a Thoma’s chamber to estimate cell viability and concentration after cell isolation.

Ten million chicken B cells per mL were seeded in 400 mL of the lymphocyte culture medium in 150 cm^2^ flasks. Chicken B cells flasks were individually infected at a multiplicity of infection (MOI) of 0.001 with each viral strain. The infected cells were maintained at 40 °C in a humidified 5% CO_2_ incubator for 48 h. Flasks content were then centrifuged at 1500 *g* for 4 min (4 °C) to pellet cell debris and supernatants were recovered. The latter were transferred to Amicon Ultra-15, PLHK, membrane Ultracel-PL, 100 kD (reference UFC910024, Merck-Millipore) and centrifuged 45 min at 3500 *g* (4 °C) in order to eliminate as much as possible proteins and to obtain high-titer viral stocks (80-fold concentration capability). Concentrated supernatants were recovered and stored at –80 °C.

### Viral titration revealed by immunochemistry (ICC)

Ten-fold serial dilutions of viral stocks in IMDM were distributed into 96-well U bottom plates (50 µL/well, eight replicates per viral dilution). Freshly prepared bursal cells in lymphocyte culture medium were added in each well (10^6^ cells in 150 µL/well) and incubated at 40 °C for 48 h in a humidified 5% CO_2_ incubator. Forty-eight hours post-infection, the cells were washed with PBS and pelleted twice by mild centrifugation, then were fixed with ethanol and acetone solution (1:1 volumetric ratio) at −20 °C for at least 30 min. After removal of the fixation solution, the plates were air-dried under a chemical hood and processed immediately or stored at −20 °C until further processing. The plates were subjected to ICC as previously described [18].

Viral titers expressed as Log_10_(TCID_50_)/mL (Tissue Culture Infectious Dose) or Log_10_(TCID_50_)/g (depending on the nature of the tissue from which the viral particles were extracted) were determined using the Reed and Muench formula [19].

### Next-generation sequencing (NGS) of viral stocks

In order to assess the purity of the viral stocks used for this study, the viral stocks were sequenced using NGS. RNA from 150 µL of each viral stock were extracted using the QIAamp viral RNA mini kit (reference 52904, Qiagen) following the manufacturer’s instructions. However, linear acrylamide (reference AM9520, Thermo Fisher) at 0.025 mg/mL was used instead of carrier RNA. RNA concentration was determined by using Qubit RNA HS assay kit (Invitrogen, Q32852) on the Qubit^®^2.0 Fluorometer.

For RNA sequencing, NGS was performed on the RNA extract after ribosomal RNA (rRNA) depletion with NEBNext rRNA Depletion Kit (NEB), as described by the manufacturer. A RNA library was obtained using Ion total-Seq Kit v2 (Life Technologies) according to the manufacturer’s recommendations and was then sequenced using Ion Torrent Proton technology. Reads were cleaned with the Trimmomatic (0.36) software (ILLUMINACLIP:oligos.fasta:2:30:5:1:true LEADING:3 TRAILING:3 MAXINFO:40:0.2 MINLEN:36). Then a Bowtie 2 (version 2.2.5) alignment was performed (–very-fast –score-min L, −0.5, −0.2 –non-deterministic -N 1) with down-sampled reads on a local nucleotide (nt) database to identify virus references. Another Bowtie 2 alignment was performed (–very-fast –non-deterministic -N 1) using *Gallus gallus* genome against cleaned reads; unmapped reads were extracted with samtools (1.8). The IBDV references with the highest number of matching reads were used for an alignment with bwa mem (0.7.8) against unmapped reads. The reads from this third alignment were collected then down-sampled to fit a global coverage depth estimation of 80 × and were submitted to the SPAdes (3.10.0) de novo assembler and Mira (4.0.2) de novo assembler (related raw reads for mira). The de novo contigs were then submitted to Megablast on the local nt database.

The best matching sequences (selected using their accession number) were used as references for a bwa mem alignment. Finally, the de novo assemblies and the alignment on the references were compared and the strict identities of the de novo and aligned sequences were assessed for validation of the final sequences using the Integrated Genomic Viewer 2.8.10 program.

Kraken [20] was used in a metagenomics approach to assign taxonomic labels to all the sequences found in the samples in order to highlight the presence of adventitious agents.

### Ethical statement

All animal trials were conducted in animal facilities approved for animal experiments (n° C-22–745–1); chickens were raised and humanely euthanized in agreement with EU directive number 2010/63/UE. Pathogenicity assessment in SPF chickens was approved by ANSES ethical committee, registered at the national level under number C2EA-016/ComEth ANSES/ENVA/UPEC and authorized by French Ministry for higher education and research under permit number APAFiS#4945-20 16041316546318 v6.

### Animal experiments: experimental design

A first experiment was designed to characterize, under standardized experimental conditions, the pathogenicity of the five pathogenic viruses for 21 days post-inoculation (dpi) (Experiment 1). This experiment was complemented by two additional experiments designed to study the early responses to infection by attenuated (Experiment 2) or pathogenic (Experiment 3) IBDV strains. Previous experiments performed in our laboratory revealed a peak in virus cloacal shedding at 2 dpi and blood B cell depletion at 4 days post-infection by a very virulent strain [21]. Therefore, 2 dpi (time of clinical signs onset), and 4 dpi (peak of clinical signs), were chosen as experiments 2 and 3 time points.

Three-week-old SPF White Leghorns chickens (ANSES, Ploufragan, France) were distributed into groups of similar weight and sex, housed in separate negative-pressure filtered-air isolators (except for the mock-inoculated groups which were housed in separate positive-pressure isolators), as presented in Table 2. Three days before inoculation, blood samples were collected from one third of the flock in order to confirm seronegativity against IBDV using a viral neutralization assay, as previously described [22]. Viral inocula were prepared by diluting viral stocks in PBS supplemented with penicillin (200 IU/mL), streptomycin (0.2 mg/mL) and fungizone (2 mg/mL). Chickens in the infected groups were inoculated by the intranasal route with 0.1 mL of virus (10^6^ TCID_50_/mL, equivalent to 10^5^ EID_50_/chicken). Mock chickens were mock-inoculated with diluent.

**Table 2:** **Experimental design.**

Experiment	Inocula	Duration (dpi)	Birds per group	Follow-up
1 Pathogenicity assessment	Vv1, Vv2, Cla	21	25	- Daily clinical score up to 10 dpi
	Im1, Im2, Mock		20	
2 Evaluation of new parameters	Vv1, Vv2, Cla	4	25 (10 animals were euthanized at 2 dpi and the remaining animals were used at 4 dpi: 10 for Cla, 11 for Vv1 and Vv2 groups)	- Blood samples on tubes with EDTA: Total blood cell count at 2 and 4 dpi - Bursa samples: Bursa to body weight ratios at 2 and 4 dpi and histological analysis -Spleen samples: Spleen to body weight ratios at 2 and 4 dpi - Blood samples on serum tubes: Uric acid dosage - Daily clinical score up to 4 dpi
	Im1, Im2, Mock 2		20 (10 of them were euthanized at 2 dpi and the remaining animals were used at 4 dpi: 10 for Im1, Im2 and Mock groups)	
3 Evaluation of new parameters	i vaccine, i + vaccine, Mock 1	4	20 (10 of them were euthanized at 2 dpi and the remaining animals were used at 4 dpi: 10 for i vaccine, i + vaccine and Mock groups)	- Blood samples on tubes with EDTA: Total blood cell count at 2 and 4 dpi - Bursa samples: Bursa to body weight ratios at 2 and 4 dpi and histological analysis -Spleen samples: Spleen to body weight ratios at 2 and 4 dpi - Blood samples on serum tubes: Uric acid dosage - Daily clinical score up to 4 dpi

### Clinical and pathological follow-up

Mortality rates were followed throughout the animal experiments. Clinical monitoring was performed from day 0 to day 10 (Experiment 1) or until termination of the experiment at day 4 (Experiments 2 and 3). Clinical signs were measured daily based on a symptomatic index (Additional file 2) previously developed, which ranges from 0 to 3 with increasing severity, an index of 3 representing the ethical endpoint of the experiment [23]. At the end of the experiments, all remaining chickens were weighed, humanely euthanatized, necropsied and their spleens and BFs were collected and weighed for calculating the spleen-to-body-weight ratio (SBR) and the bursa-to-body-weight ratio (BBR), respectively.

### Histopathological analysis and bursal lesions scoring (Experiments 2 and 3)

Two to three tissue samples of bursa per day and per group stored in a 75% ethanol solution were analyzed by a pathologist (Labocea, Ploufragan, France) to score the histopathological lesions according to Skeeles et al. [24].

### Determination of the viral load in the bursa at 2 and 4 dpi (Experiments 2 and 3)

At 2 dpi, for the groups infected with Vv1, Vv2 and Cla viruses (Experiment 3), ten chickens of each group were selected according to their high clinical score. In the mock, im1 and im2 viruses infected groups (Experiment 2), 10 chickens of each groups were randomly chosen, weighed and humanely euthanatized. During necropsy (see above), a piece of bursa was collected and processed for viral titration. At 4 dpi, the same procedure was repeated on all the remaining chickens. Viral particles from each 2 and 4 dpi collected BF were extracted as follows. Bursal tissue was homogenized using Tissue Lyser (Qiagen) homogenizer to process individual BFs. All steps were carried out on ice. Briefly, bursae were weighed and cut into small fragments. PBS was added (1 mL of PBS per gram of bursa) to the chopped bursa before homogenizing with a stainless steel bead for 3 min at 30 Hz with a Qiagen Tissue Lyser. 1, 1, 1, 2, 3, 4, 4, 5, 5, 5-Decafluoropentane (reference 94884, Sigma) was added to the tissue suspension (at an approximate 1:1 volumetric ratio) and an additional homogenization step was carried out followed by centrifugation at 4000 *g* for 30 min. Supernatants were collected and stored at −80 °C for a later titration using the above-described ICC.

### Blood cell counts using flow cytometry (Experiments 2 and 3)

At 2 and 4 dpi, chickens were blood sampled at the venous occipital sinus before euthanasia, using commercial ethylenediaminetetraacetic acid (EDTA) coated blood collection devices (S-Monovette EDTA K2 2.7 mL, Sarstedt, reference 04.1915.100). Previous observations from the authors’ laboratory determined that jugular sampling during euthanasia, even with EDTA coated collection devices, led to significant coagulation of the samples. All blood samples were kept at room temperature and processed within 4 h after blood collection according to Seliger et al. [25]. Briefly, blood samples were diluted in staining buffer (PBS with 1% FBS), mixed with the labeled antibody mixture and incubated for 30 min with agitation (500 revolutions per minute or rpm) at room temperature and in the dark. The antibodies and fluorochrome conjugates used in this study and their dilution before use are indicated in Additional file 1. After incubation, Precision Count Beads (reference BLE424902, BioLegend) prepared in staining buffer were added to each sample to determine the absolute counts of cells. To inactivate the virus, formaldehyde (1% final concentration) was added and the samples were incubated for 15 min with frequent agitation (500 rpm) at room temperature and in the dark to inactivate the virus [26]. Samples were analyzed on a FC500 MPL flow cytometer (Beckman Coulter), with a previously used gating strategy [21]. The results were transformed by the logarithmic function for the rest of the analyses. The application of this transformation ensures the normal distribution of the variable studied (especially if there are outlier values among the results). Cell concentrations below the level of detection of the flow cytometer leading to a result of “0 cells/µL” were manually replaced with “1 cell/µL” to allow analysis of the logarithmic results since the value log_10_(0) is not defined.

### Uric acid dosage (Experiments 2 and 3)

Dosage of uric acid was performed individually on mock- and virus-inoculated animals whose sera had been collected on serum tube. The Cayman uric acid titration kit (Cayman Chemical, 700320) was used according to manufacturer’s instructions. Briefly, 15 µL of each animal serum was mixed with 105 µL of diluted Assay Buffer, 15 µL of Fluorometric Detector and 15 µL of Enzyme Mixture. The mix was incubated for 15 min at room temperature. The fluorescence was measured using a Tecan Infinite M200 Pro.

### Statistical analyses

Our first aim was to classify the very virulent, virulent (comprising Cla, im1 and im2 strains) and attenuated (i + vaccine and i vaccine) pathotypes of the strains with respect to 9 explanatory variables: the 7 blood concentrations (i.e., logarithms of concentrations of B cells, T cells, monocytes, granulocytes, erythrocytes, thrombocytes, uricemia), the bursal viral load and the clinical score. For this purpose, 182 observations were available, corresponding to 90 chickens at 2 dpi and 92 chickens at 4 dpi.

All datasets from experiments 2 and 3 at 2 dpi were arranged in a multivariate manner with animals in rows and, as columns, variables measured during the experiments: logarithmic blood concentrations of B cells, T cells, monocytes, granulocytes, erythrocytes and thrombocytes, logarithmic uricemia, logarithmic bursal viral load, clinical score at 2 dpi and group of infection. The same approach was applied to the data at 4 dpi in a different dataset.

A factorial discriminant analysis was applied to illustrate the differences between pathotypes and associate them with the explanatory variables under study [27]. All statistical analyses were performed using R (version 4.0.3) [28]. This analysis required the R packages data.table (version 1.14.2) [29], FactoMineR (version 2.4) [30], factoextra (version 1.0.7) [31], gridExtra (version 2.3.) [32], missMDA (Version 1.18) [33] (missing values imputation was performed), corrplot (version 0.92) [34] and the function discrimin of the ade4 package [35].

Then, a machine learning procedure was used to evaluate the predictive performance of ten classification models, the discriminant analysis being one of these models, and to select the model with the best predictive performances. The caret R package was used [36]. The ten selected models were: naïve Bayes classifier [37] (Version 0.9.7), Weighted k-Nearest Neighbors Classification (Version 1.3.1) [38], Random Forest (Version 4.7-1) [39], Kernel method (Version 0.9-29) [40], Neural networks (Version 0.4-14) [41], Bagged tree (Version 6.0-90) [36], C5.0 (Version 6.0-90) [36], Multi-Layer Perceptron (Version 6.0-90) [36] and L2 Regularized Support Vector Machine (dual) with Linear Kernel (Version 6.0-90) [36]. A repeated (100 times) two-fold cross-validation procedure (i.e., 70% of the observations = training dataset; 30% of the remaining observations = test dataset) allowed to assess the predictive performance of each of the ten models under study, which corresponded to the percentage of the well-classified observations. The required parameters of each model were tuned by means of a repeated (10 times) tenfold cross validation procedure applied to the training dataset. The global performance, or accuracy, of the models was evaluated throughout the percentage of animals correctly assigned to an infected group (for example, among all infected chicken, how many of them were correctly assigned to their infected group). A pathotype-specific performance of each model was also evaluated throughout the percentage of animals infected with a strain of a defined pathotype accurately classified (for example, among all the Cla infected chickens, how many of them were correctly assigned to the Cla infected group).

Our second aim was to describe the individual variations of the valuable parameters selected in the final model. Differences in percentages of mortality were analyzed using Fisher’s exact test followed by pairwise comparisons using the fisher.multcomp function from RVAideMemoire package version 0.9-79 [42]. All other quantitative parameters were analysed using Kruskal–Wallis test followed by Fisher’s least significant difference test with Holm adjustment method for multiple comparisons using the kruskal function from the Agricolae package version 1.3-3 [43].

## Results

### Purity of the viral stocks

The viral stocks used to inoculate the animals presented eukaryotic, viral and bacterial reads. The highest percentages of reads for each viral stock were eukaryotic reads (most of them assigned to *Gallus gallus*)*.* The viral reads were all assigned to IBDV taxon.

### Confirmation of the pathotypes of viral strains after propagation on B cells

#### Clinical signs

**Figure 1:**
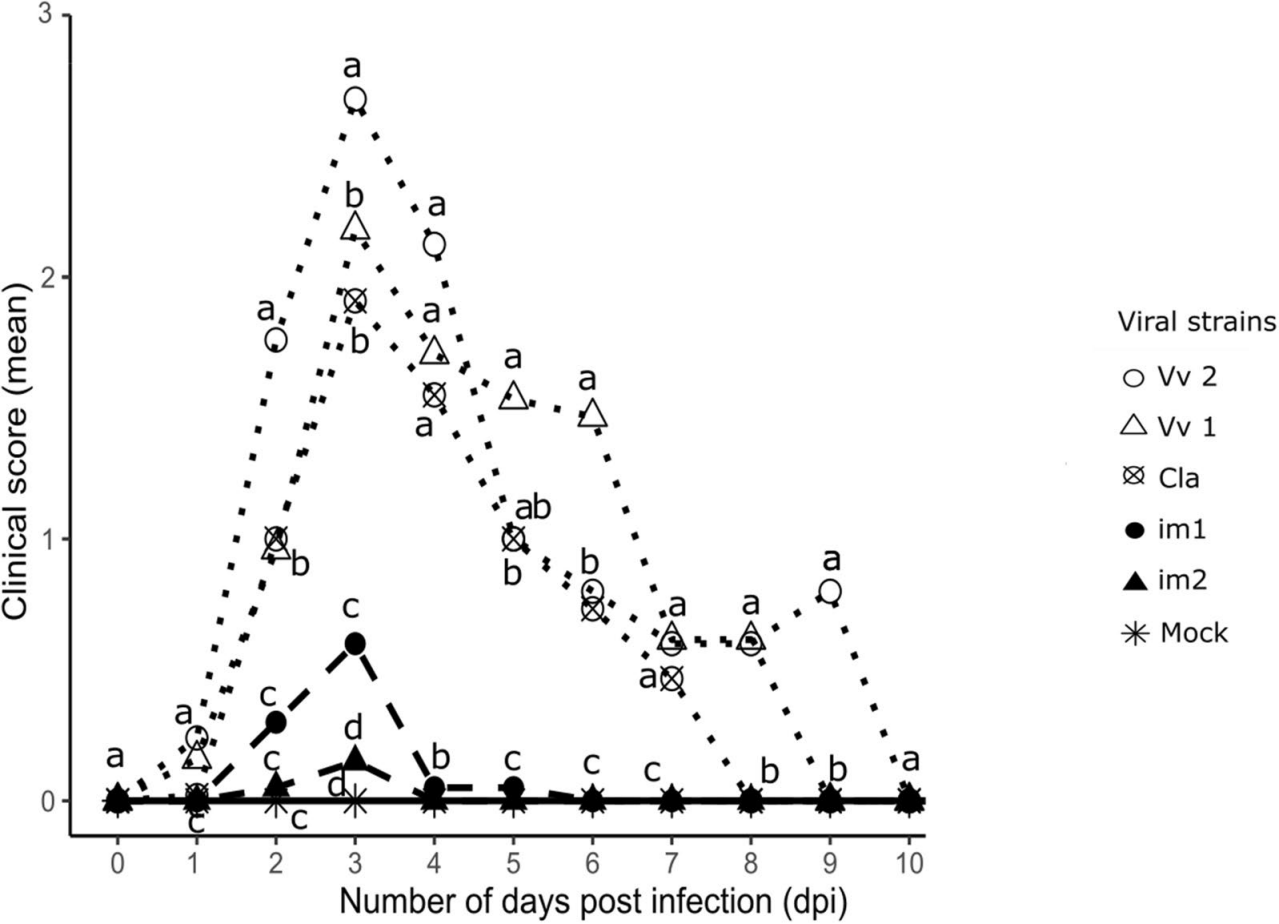
**Evolution of clinical signs on 10 days (experiment 1), using the symptomatic index as described in Additional file **2**.**

As presented in Figure 1, mock-infected animals did not develop any clinical sign during experiment 1. For infected groups, clinical signs were first visible at 1 dpi, then reached a peak at 3 dpi. All surviving animals recovered by 10 dpi. At 3 dpi, the mean clinical score was 0.2 for im1, 0.6 for im2, 1.9 for Cla, 2.2 for Vv1 and 2.7 for Vv2 strain.

The study of the mortality rate (Experiment 1) revealed that mock-infected group as well as im1 and im2 infected groups did not experience any mortality. The Cla infected group experienced 40% mortality, which was not significantly different from the mortality observed in the Vv1 group (48% mortality, *p* = 0.97). This mortality rate was significantly lower than the 80% mortality observed in the Vv2 group (*p* = 0.014).

#### Gross lesions

The necropsies performed in experiments 2 and 3 did not reveal any lesions in mock and attenuated groups at 2 and 4 dpi. At 2 dpi, 10% of the animals of the Cla and Vv2 infected groups showed at least one type of lesions of the bursa (apart from oedema and atrophy which are presented below) and 20, 20, 30, 40 and 50% of animals from im1, im2, Cla, Vv1 and Vv2 groups, respectively, showed at least one type of spleen lesion. Bursal and muscular haemorrhages were specifically observed in Cla (10 and 70% respectively) and Vv1 groups (10 and 90% respectively). At 4 dpi, 28, 36 and 9% of the animals of the Cla, Vv1 and Vv2 respectively showed at least one type of bursal lesions and 91, 91, 91, 72, 90% of the animals of the im1, im2, Cla, Vv1 and Vv2 groups respectively showed at least one type of spleen lesion. Bursal and muscular haemorrhages were specifically observed in Cla (25 and 9% respectively), Vv1 (36 and 55% respectively) and Vv2 (91 and 9% respectively) groups (Additional file 3 and Additional file 4).

#### Microscopic lesions

The Bursa of Fabricius lesions score (BLS) was 0 for all mock-inoculated animals at 2 and 4 dpi. Even if some bursas from animals of the i group showed mild level of lesions, there was no significant statistical difference between this group and the mocks at 2 and 4 dpi. The BLS globally increased with the pathogenicity of the virus at 2 and 4 dpi. There was no significant statistical difference between the Cla and very virulent infected bursa (with BLS of 4) at 2 and 4 dpi (Additional file 5).

#### BBR

At 2 and 4 dpi, BBR in the mock-infected groups of experiments 2 and 3 were similar and ranged from 4.3 to 4.6‰. Despite no statistically significant difference between any group BBR and their respective mock (beside im1), early edema was observed at 2 dpi on 10 to 30% of the animals of the infected groups (Additional file 6).

At 4 dpi, among the infected groups, only the i + vaccine, im1, im2 and Vv2 infected group showed a statistically lower BBR than their mock-inoculated controls. A slight atrophy was observed at 4 dpi, with a tendency towards more severe atrophy for the im1 and im2 strains.

#### SBR

At 2 dpi, only the i + vaccine infected group showed a statistically higher SBR than its mock-inoculated control (1.8‰). Other groups SBR ranged from 1.2 to 1.5‰.

At 4 dpi all the infected groups, apart from the one infected by the i vaccine, showed statistically higher SBR than their mock’s with median values ranging from 2 to 4.6‰.

Collectively, the observed clinical score, mortality and lesions are consistent with the expected pathotype of the strains used (Additional file 7).

### Identification of key discriminant factors of the pathotype through machine-learning methods

**Figure 2:**
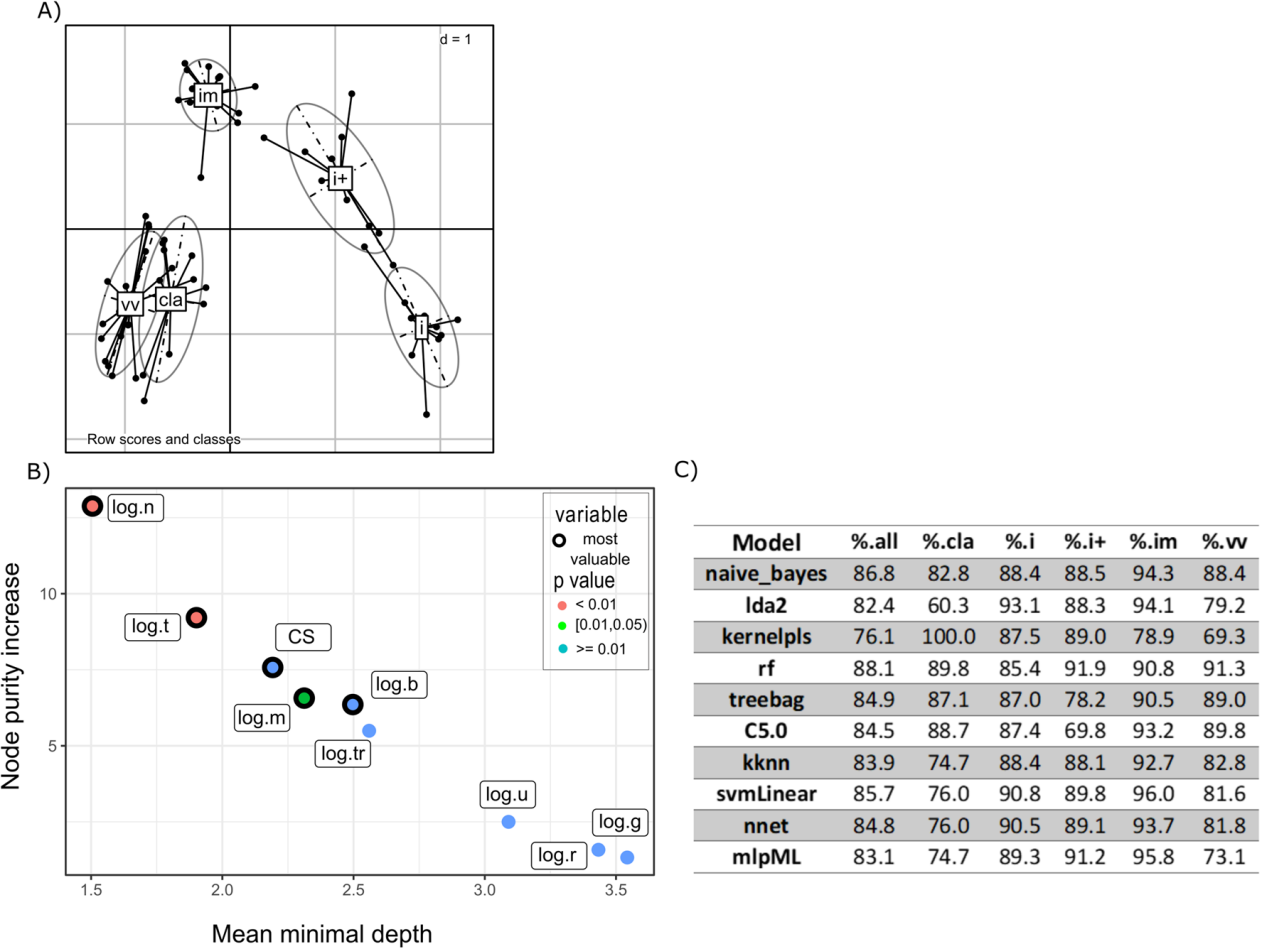
**Based on data collected at 2 dpi**. **A** Factorial discriminant analysis (FDA) projection of each individual onto the first two components (43 and 18% of the data inertia). Lines link individuals to the centre of gravity of their infecting pathotype group and are associated with their confidence ellipse. Cla gathered the animals infected by the Cla strain, im those infected by im1 or im2 strains, i those infected by i vaccine strain, i + those infected by i + vaccine and Vv those infected by Vv1 or Vv2 strains, **B** Explanatory variable importance plot considering node purity increase and mean minimal depth [44] for the random forest model associated, **C** Performances of models in terms of percentages of correctly classified individuals (r:  erythrocytes blood concentration, thr: thrombocytes blood concentration, m : monocytes blood concentration, g:  granulocytes blood concentrations, b: B lymphocytes blood concentration, t: T lymphocytes blood concentration, n:  bursal viral load, u: uric acid blood concentration, CS: clinical score), d: dimension.

**Figure 3:**
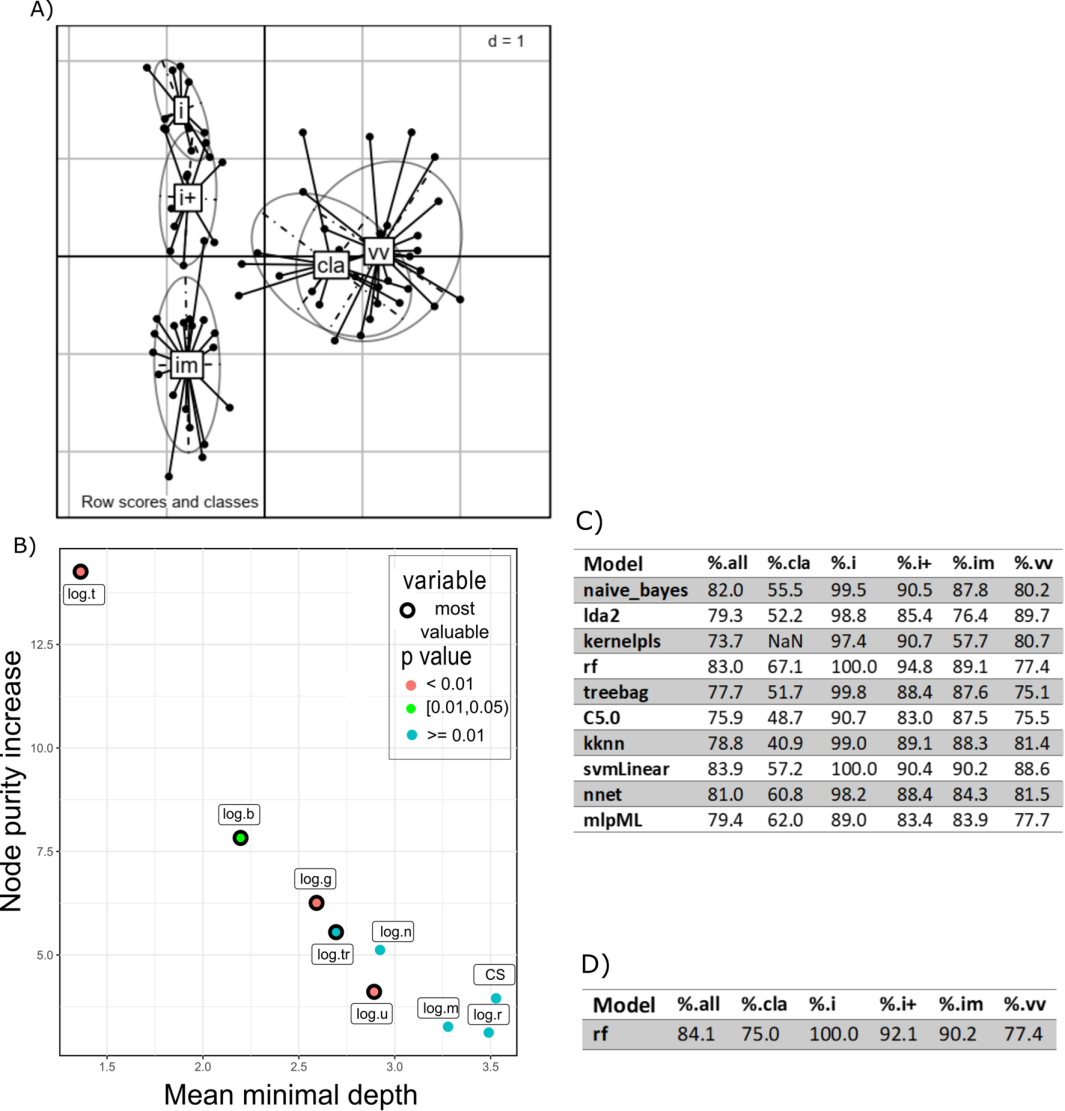
**Based on data collected at 4 dpi. A** Factorial discriminant analysis (FDA) projection of each individual onto the first two components (43 and 18% of the data inertia). Lines link individuals to the centre of gravity of their infecting pathotype group and are associated with their confidence ellipse. Cla gathered the animals infected by the Cla strain, im those infected by im1 or im2 strains, i those infected by i vaccine strain, i + those infected by i + vaccine and Vv those infected by Vv1 or Vv2 strains, **B** Explanatory variable importance plot considering node purity increase and mean minimal depth [44] for the random forest model associated, **C** Performances of models in terms of percentages of correctly classified individuals (r: erythrocytes blood concentration, thr: thrombocytes blood concentration, m: monocytes blood concentration, g : granulocytes blood concentrations, b:  B lymphocytes blood concentration, t:  T lymphocytes blood concentration, n:  bursal viral load, u:  uric acid blood concentration, CS:  clinical score), d:  dimension.

Factorial discriminant analysis was conducted on the clinical score, bursal viral load, uricemia and cell blood concentrations (B cells, T cells, monocytes, granulocytes, thrombocytes and erythrocytes) at 2 and 4 dpi, to determine if any combination of these parameters would discriminate the different pathotypes infecting the different groups. The factorial discriminant analysis (FDA) projection onto the first two components showed that animals were grouped together based on their experimental groups. Furthermore, each group corresponding to each pathotype was rather well separated from the others, with partial overlapping for the Cla, Vv1 and Vv2 groups on the one side and the i vaccine and i + vaccine groups on the other side (Figures 2A and 3A).

Machine learning procedure was then used to establish predictive models using those parameters. The performance of the models (percentage of animal classified in the correct pathotype group based on the parameters) are presented in Additional file 8 and Additional file 9 and for both 2 and 4 dpi. Our criteria to select a model were a global performance value above 80% and specific performance values above 65%. Even if the global performance of the svmlinear was the best, the Random Forest was the best model based on its specific predictive performance. Performance analysis of those two Random Forest models determined the most valuable parameters for each one (Figures 2B and  3B) using Random ForestExplainer [44]. Detailed accuracy brought by each parameter to the 4 dpi model into determining each pathotype is available (Table 3). The performance of the Random Forest models only relying on the most valuable parameters was then measured (Figures 2C and 3C). At 2 dpi, the bursal viral load (n), clinical score (CS) and the blood concentrations of B cells (b), T cells (t) and monocytes (m) were sufficient to established a model with a global performance above 80% and specific performances above 65%, matching our threshold requirements. At 4 dpi, the most valuable parameters (T cells, B cells, granulocytes (g) and thrombocytes (tr) blood concentrations and uricemia) allowed to establish a model with a global performance still above 80% but with lower performance for the classification of animals infected by Cla strain (specific performance of 67.1%). Different sets of parameters were used then to establish Random Forest models and determine if one peculiar set could lead to a better specific classification including Cla strain infected animals. The Random Forest model established only on the blood concentrations parameters showed a better performance toward Cla strain classification and a global performance above 80% (Figure 3D). The performance of this predictive model differed between pathotypes. The lowest prediction performances were associated with the Cla, Vv1 and Vv2 strains (Additional file 10).

**Table 3:** **Mean decrease in accuracy of the established random forest model if removing the variable.**

	Classical pathotype (Cla strain)	Highly attenuated pathotype (i vaccine strain)	Attenuated pathotype (i + vaccine strain)	Strictly immune-suppressive pathotype (im1 and im2 strains)	Very virulent pathotype (Vv1 and Vv2 strains)
Clinical score at 4 dpi	0.02	0.06	0.05	0.07	0.03
Log (r)	0.03	0.03	0.03	0.03	0.00
Log (thr)	−0.01	0.03	0.08	0.04	0.10
Log (m)	0.06	0.00	0.00	0.00	0.04
Log (g)	0.06	0.07	0.15	0.02	0.04
Log (b)	0.02	0.45	0.12	0.04	0.03
Log (t)	0.06	0.08	0.01	0.44	0.17
Log (n)	0.01	0.25	0.01	0.04	0.01
Log (u)	−0.02	−0.01	0.13	0.01	0.01

Features analysis was performed using the RandomForestExplainer [44].

r : erythrocytes blood concentration, thr:  thrombocytes blood concentration, m: monocytes blood concentration, g: granulocytes blood concentrations, b: lymphocytes B blood concentration, t: lymphocytes T blood concentration, n: bursal viral load, u: uric acid blood concentration at 4 dpi.

Based on the chosen models, bursal viral loads, 2 dpi total blood count and uric acid blood concentration were not key discriminant factors for pathotyping IBDV strains, at least those included in this study. Thus, their associated results are available in Additional file 11, Additional file 12, and Additional file 13, respectively. However, it is interesting to mention that at 4 and especially 2 dpi, the viral load in the bursa showed strong disparities between the strains and the pathotypes.

### Pathotype-specific early changes of the blood cell formula

**Figure 4:**
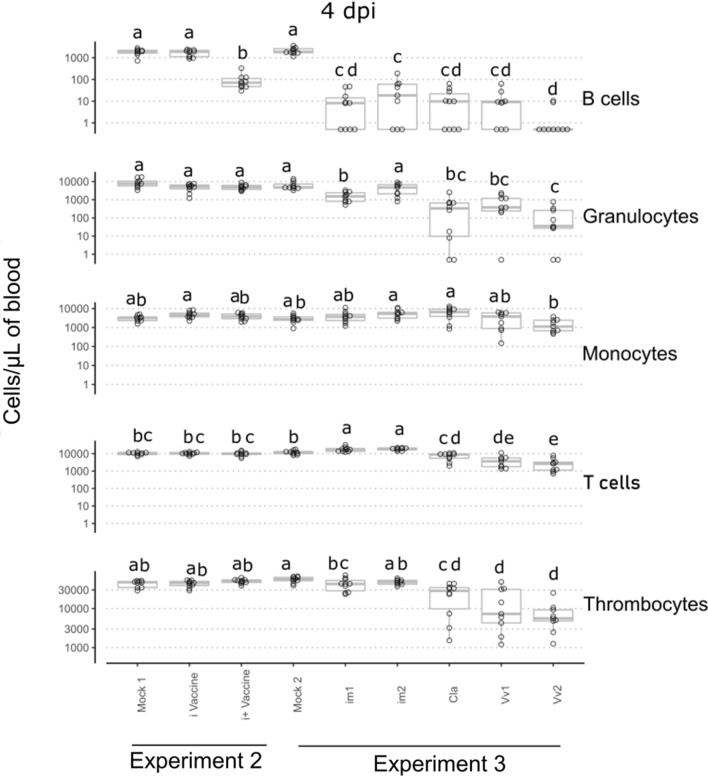
**Concentrations of blood cells at 4 days post-infection (groups with at least one letter in common did not show any significant statistical variation of their median value).**

The blood concentration of erythrocytes did not vary between mocks and infected groups (Additional file 14). As presented in Figure 4, infection by the i vaccine did not induce any significant statistical variation of the blood B cells concentration compared to the mock. In contrast, the i + vaccine induced a slight but significant decrease of B cells concentration compared to the mock and im1, im2, Cla, Vv1 and Vv2 induced a massive drop of that concentration. Only im1 and im2 strains induced an increase of the T cell concentration while Cla, Vv1 and Vv2 were associated to a decrease of this cell type compared to the mock. The Cla, Vv1 and Vv2 strains induced a specific severe thrombocytopenia and decrease of granulocyte concentration. Vv1 and Vv2 were the only strains to induce a slight decrease of the monocyte concentration.

## Discussion

The aim of this study was to provide a proof of concept of an alternative protocol for pathotyping IBDV strains. Although genetic analysis performed on both genomic segments [9] may indicate a possibly altered virulence, for instance if reassortment is observed, as seen in our virus panel with strains Vv1 (genotype A3B2) and Vv2 (genotype A3B3), in vivo pathotype determination remains necessary to ascertain such impact.

The goal of experiment 1 was to confirm the pathotype of the IBDV strains included in our panel using the traditional pathotyping protocol. Clinical monitoring was globally consistent with expectations. Very virulent strains usually induce a mortality twice superior to classic virulent ones [10] but in this experiment, we unexpectedly observed very close mortalities for the classic virulent and Vv1 strains. This illustrates the variability inherent to in vivo experiments on which the pathotyping of IBDV is currently based, especially when relying on single experiments. As it relies on parameters such as mortality and clinical score, whose values may easily vary between laboratories or even vary when repeating the assay within the same laboratory for the same strain, such a system cannot be perfectly standardized [45]. This might explain why CS at 4 dpi was not selected as a valuable variable to predict the pathotype in our model. Simultaneous study of a panel of viruses belonging to all known pathotypes in experiments 2 and 3 suggests that other quantitative parameters could be used more reliably and combined in a predictive model possibly predicting the pathotype.

The new pathotyping protocol presented in this article addresses several issues associated with the traditional one. Experiments 2 and 3 analyze time points aimed at providing early insights into IBD physiopathology. Our results revealed that 4 dpi blood concentrations are required and possibly sufficient to establish a predictive model at the base of a potentially faster, simpler and more ethical protocol of pathotyping. The reduced number of animals required by the machine learning makes this model a protocol in line with the prevailing 3R approach. The small number of animals (from 10 to 11 per group) used in our experiments (Table 2) was sufficient to establish a model with high performance of prediction for all pathotypes. This new protocol thus allows to reduce the number of animals used since traditional pathotyping, relying only on mortality, would need for example at least 23 animal per group to show, with α = 0.05 and β = 0.2, a statistically significant difference between the mortality induced by Vv2 and Cla strains in experiment 1 (number determined using the power.prob test function of R software). Further experiments will be needed to determine the minimal number of animals required for pathotyping IBDV using the new protocol.

Even though our results revealed that a model might be established based on data obtained at 2 dpi, it was not considered the best alternative to the traditional pathotyping protocol because of viral titration, which is a rather time-consuming and constraining method.

Regarding the limitations of this new protocol, two major ones are to be mentioned: the use of the White Leghorn chicken breed and the SPF status of the animals. Different sensitivity and responses to IBDV infection have been reported between chicken breeds [4, 46], partially relying on a differential bursal T cell response, one of the valuable blood parameters used in the machine learning process. The model performance and relevance could be modified when using another chicken breed, such as commercial meat-type chickens (notwithstanding the possible presence of anti-IBDV maternally derived antibodies in such commercial chickens). Relying on blood cell concentration to identify a pathotype requires that the observed variations can be attributed reliably to infection by IBDV. As an example, it has been shown that total blood count values can be influenced in non-infected animals by their age and their genetic background [25]. The chickens used here were inoculated with IBDV viral stocks whose purity was assessed by NGS. In the field, co-infection by IBDV with other viruses like chicken anemia virus [47] are often observed. These other agents can themselves induce changes in the organs and blood cells concentrations, like the chicken anemia virus that induces immunosuppression by destroying T lymphocytes [48, 49]. Identifying the true causal agent responsible for the observed blood cells concentrations changes in an animal co-infected by such agents would not be possible. This model might then only allow the pathotyping of pure IBDV strains in a fully controlled animal experiment using SPF chicken. These are however the exact limitations of the traditional method too.

Furthermore, the data used to supply the machine learning algorithms contained a limited number of strains for each pathotype. Including more variant and reassortant IBDV strains would enhance the model relevance regarding the pathotyping of recently appeared strains of that kind [50–52].

As mentioned in the results, the performances of the predictive model for the classic virulent and very virulent pathotypes are low compared to the other pathotypes. This residual variability might be explained by the fact that two variables of major importance for our model are the concentrations of thrombocytes and granulocytes. Since analysis of the individual values of these variables reveals the presence of outliers with similar values for both pathotypes. This similarity could explain a certain degree of model confusion that would misclassify a classic virulent inoculated individual as a very virulent inoculated one and vice versa, leading to a lower degree of predictive performance. It could be hypothesized that the pathogenesis processes behind infection with a classic virulent or hypervirulent virus are similar but different in terms of the percentage of individuals affected.

Having defined new required and sufficient key parameters at 4 dpi to pathotype an IBDV strain as the blood concentrations of B cells, T cells, monocytes, granulocytes, thrombocytes and erythrocytes, it is interesting to investigate tendencies for pathotype-specific variations at 2 and 4 dpi, as an insight into the pathogenesis of the different IBDV pathotypes.

Variations in the concentration of blood cells may be explained by disruption of cell production, cell destruction or cell migration from the blood stream to the tissues. Such a variation in blood cell populations, in particular blood lymphocytes, has been linked previously to an infectious context, leading to a transient lymphopenia through differential redistribution of cells [53]. However, the causal link between the recruitment of cells to an organ and the reduction in their blood concentration has not yet been established.

Previous studies demonstrated the connection between IBD infection and a decrease of blood erythrocytes concentrations when our data suggested otherwise [54]. Since the purity of the IBDV viral stocks used in this article might not have been assessed like in ours and given that their method for measuring the blood cells concentrations differs from ours, we can hypothesize that the divergence of conclusions among the two studies may be caused by those two elements.

We observed a near disappearance of blood B cells at 2 dpi with pathogenic strains and a bursal atrophy tendency at 4 dpi. This is consistent with the fact that IBDV infection targets proliferating B cells [55] and induces massive lesions of the bursa [56]. A drop of the B cells blood concentration after infection by a virulent strain, such as the one observed in the current study, is consistent with observations made by our team [21] and others [57]. Indeed, an early and massive reduction in B cell counts was observed after the infection of three-week-old chickens by IBDV. The i vaccine induced no decrease of B cells and the i + vaccine only a slight decrease. Such an observation is consistent with the previous observation made by our team [58] on the correlation of blood B cell depletion with the level of attenuation of IBDV vaccines.

As for the T cells, a global transient decrease of their blood concentration at 2 dpi was observed. However, even if the T cell concentration tends to demonstrate the same tendency for almost all groups, a different pattern was observed for im1 and im2 strains. Those strains were associated with T cell concentrations higher than those of the mock group. The absence of inflammatory response of animals infected by strictly immunosuppressive strains has been shown previously [59]. Recruitment of immune cells to the infection site has been observed in various diseases, like with IBDV [16], through a change of bursal cell population nature with an entry of T cells in the bursa from 4 dpi which is consistent with the blood population decrease we observed. The important role of intra bursal T cells has been linked to viral clearance [60]. Thus, we might speculate that the decrease of blood T cells might be due to a recruitment of those cells on the infection site through the secretion of inflammation factors.

The granulocyte concentration, showing a slight increase tendency at 2 dpi, showed a decrease at 4 dpi for the Cla, Vv1 and Vv2 strains. The blood granulocyte decrease phase observed here resonates with bursal infiltration studies conducted on IBDV infected chicken that revealed a granulocyte infiltration of the bursa at 3 dpi [61].

In our study, thrombocytopenia was specifically observed with Cla, Vv1 and Vv2 strains, it was slight at 2 dpi and severe at 4 dpi. This appears to be consistent with the specific muscular and bursal haemorrhages observed during necropsy. Data in the present paper are consistent with previous studies which linked coagulation abnormalities (in particular an increase in coagulation time) and the severity of IBD [62], and with more recent observations of an increase of the prothrombine time after IBDV infection [63]. This impact on the coagulation cascade might explain clinical outcome or gross pathology differences behind the different pathotypes. Furthermore, the avian thrombocytes have been shown to express toll-like receptors [64] and produce cytokines [65]. Their role in the pathogenicity of avian diseases has been documented for avian influenza [66] but not demonstrated yet for IBD [67].

Pathotype-specific differences in total blood count, as observed in the present study, support a pathotype-specific pathogenesis model based on differential recruitment of immune cells in the immune organs or differential destruction of the immune blood cells.

Beyond the scope of this study, investigation of the origin or outcome of the decrease in blood cells concentrations and their exact subset might deepen the current limited knowledge of the mechanisms underlying the pathogenesis of the different IBDV pathotypes. Assessing the level of immune cell infiltration in the bursa and spleen during infection could possibly help in addressing the question behind the pathotype-specific variations in blood cells concentrations.

As a conclusion, the wealth of information brought by blood immune cells counting during IBDV infection paves the way to a simplified pathotyping of this virus compared with the traditional protocol. It may help to reduce the number of animals used for this purpose.

### Supplementary Information

**Additional file 1:** **Antibodies used for flow cytometry based white blood cell counting.**

**Additional file 2:  Symptomatic index explicitation.**

**Additional file 3: Percentage of animal presenting lesions (experiments 2 and 3) at 2 dpi.**

**Additional file 4:** **Percentage of animals presenting lesions (experiments 2 and 3) at 4 dpi.**

**Additional file 5: Mean bursal lesions score of different groups on the same day (groups with at least one letter in common did not show any significant statistical variation of their median value).**

**Additional file 6: Bursa body weight ratio at 2 and 4 dpi (groups with at least one letter in common did not show any significant statistical variation of their median value).**

**Additional file 7: Spleen body weight ratio at 2 and 4 dpi (groups with at least one letter in common did not show any significant statistical variation of their median value).**

**Additional file 8: Models performance at 2 days post-infection with the following parameters taken into account**: bursal viral load, uricemia, blood cells concentrations (all), clinical score. Cla gathered the animals infected by the Cla strain, im those infected by im1 or im2 strains, i those infected by i vaccine strain, i+ those infected by i+ vaccine and Vv those infected by Vv1 or Vv2 strains.

**Additional file 9: Models performance at 4 days post-infection with the following parameters taken into account**: bursal viral load, uricemia, blood cells concentrations (all), clinical score. Cla gathered the animals infected by the Cla strain, im those infected by im1 or im2 strains, i those infected by i vaccine strain, i+ those infected by i+ vaccine and Vv those infected by Vv1 or Vv2 strains.

**Additional file 10:  Models performance at 4 days post-infection with the following parameters taken into account: uricemia, blood cells concentrations (t, b, g, tr).** Cla gathered the animals infected by the Cla strain, im those infected by im1 or im2 strains, i those infected by i vaccine strain, i+ those infected by i+ vaccine and Vv those infected by Vv1 or Vv2 strains.

**Additional file 11: Bursal viral load at 2 and 4 dpi (experiment 2 and 3) (groups with at least one letter in common did not show any significant statistical variation of their median value).**

**Additional file 12: Blood cells concentration at 2 dpi (experiments 2 and 3) (groups with at least one letter in common did not show any significant statistical variation of their median value).**

**Additional file 13: Uric acid blood concentration at 2 and 4 dpi (experiments 2 and 3) (groups with at least one letter in common did not show any significant statistical variation of their median value)**

**Additional file 14: Erythrocytes blood concentration at 2 and 4 dpi ** (no statistically significant difference between the infected groups and the mocks was observed) (groups with at least one letter in common did not show any significant statistical variation of their median value).

## Data Availability

The datasets used and analyzed during the current study are available from the corresponding author on reasonable request.
