# Supplementary material for: Infectious bursal disease virus: predicting viral pathotype using machine learning models focused on early changes in total blood cell counts
Source: Vet Res. 2023 Oct 30;54:101. doi: 10.1186/s13567-023-01222-5 (PMC10614337; doi:10.1186/s13567-023-01222-5)
Supplement: Supplementary file 1 — Additional file 1: Antibodies used for flow cytometry based white blood cell counting. [file 13567_2023_1222_MOESM1_ESM.docx]

| Name | Specificity | Clone | Fluorochrome | Stained cells | Reference | Clone provider and reference | In-house conjugation kit (if applicable) provider and reference | Final dilution |
| --- | --- | --- | --- | --- | --- | --- | --- | --- |
| TCRgd-FITC | chTCRgd | TCR-1 | FITC | gamma-delta T cells (gd T-cells) | (1) | TCR1  (Southern Biotech, reference 8230-02) |  | 800 |
| CD4-FITC | chCD4 | CT4 | FITC | CD4+ T cells (T-helpers cells) | (2) | CT-4  (Southern Biotech, reference 8210-02) |  | 800 |
| CD8a-FITC | chCD8a | CT8 | FITC | CD8+ T cells (Cytotoxic T-cells) | (2) | CT-8  (Southern Biotech, reference 8220-02) |  | 800 |
| Kul1 | Unknown | Kul01 | RPE | Monocytes, Macrophages | (3) | KUL01 (Southern Biotech,  reference 8420-01) | Mix-n-stain RPE  (Ozyme, reference BTM92299) | 800 |
| Bu1-AF647 |  |  | AF647 | LB | (4) | Bu-1 (Southern Biotech,  reference 8395-02) |  | 800 |
| 16-6 | chCD45 | 16-6 | APC-CF750 | Leucocytes (CD45+) | (5) | UM16-6 (Bio-rad, reference MCA2413GA) | Mix-n-Stain™ APC-CF®750T  (Ozyme, reference BTM92311) | 400 |
| K1 | Unknown | K1 | RPE | Monocytes, Macrophages, Thrombocytes | (6) | Provided by Berndt Kaspers | Mix-n-stain RPE  (Ozyme, reference BTM92299) | 400 |
