# Supplementary material for: Infectious bursal disease virus: predicting viral pathotype using machine learning models focused on early changes in total blood cell counts
Source: Vet Res. 2023 Oct 30;54:101. doi: 10.1186/s13567-023-01222-5 (PMC10614337; doi:10.1186/s13567-023-01222-5)
Supplement: Supplementary file 2 — Additional file 2: Symptomatic index explicitation. [file 13567_2023_1222_MOESM2_ESM.docx]

| Symptomatic index = Clinical score | Clinical signs |
| --- | --- |
| 0 | lack of signs |
| 1 | typical IBD signs (ruffled feathers) conspicuous in quiet bird only, the bird stimulated by a sudden change in the environment (light, noise, or vicinity of experiment observer) appears normal, motility is not reduced |
| 2 | typical IBD signs conspicuous even when bird is stimulated, dehydration is apparent, motility may be slightly reduced |
| 3 | typical severe IBD signs with prostration or death (Ethical endpoint) |
