# Supplementary material for: Infectious bursal disease virus: predicting viral pathotype using machine learning models focused on early changes in total blood cell counts
Source: Vet Res. 2023 Oct 30;54:101. doi: 10.1186/s13567-023-01222-5 (PMC10614337; doi:10.1186/s13567-023-01222-5)
Supplement: Supplementary file 4 — Additional file 4: Percentage of animals presenting lesions (experiments 2 and 3) at 4 dpi. [file 13567_2023_1222_MOESM4_ESM.docx]

|  | Bursae | | Spleen | | | Muscles |
| --- | --- | --- | --- | --- | --- | --- |
| Group | Haemorrhage | Caseum | Marbled | Deep red color | Light color | Haemorrhage |
| mock | - | - | - | - | - | - |
| i vaccine | - | - | - | - | - | - |
| i+ vaccine | - | - | - | - | - | - |
| im1 | - | - | 91 | - | 18.2 | - |
| im2 | - | - | 91 | - | 45.5 | - |
| Cla | 27.3 | - | 91 | 27.3 | 63.6 | 9.1 |
| Vv1 | 36.4 | 9.1 | 72.3 | 18.2 | 36.4 | 54.5 |
| Vv2 | 91 | - | 91 | 45.5 | 45.5 | 9.1 |
