# Supplementary material for: Infectious bursal disease virus: predicting viral pathotype using machine learning models focused on early changes in total blood cell counts
Source: Vet Res. 2023 Oct 30;54:101. doi: 10.1186/s13567-023-01222-5 (PMC10614337; doi:10.1186/s13567-023-01222-5)
Supplement: Supplementary file 5 — Additional file 5: Mean bursal lesions score of different groups on the same day (groups with at least one letter in common did not show any significant statistical variation of their median value). [file 13567_2023_1222_MOESM5_ESM.docx]

| dpi | Mock Expt 3 | i vaccine | i+ vaccine | Mock Expt 2 | im1 | im2 | Cla | Vv1 | Vv2 |
| --- | --- | --- | --- | --- | --- | --- | --- | --- | --- |
| 2 | 0 ^d^ | 0.5 ^d^ | 1.75 ^c^ | 0 ^d^ | 2.0 ^bc^ | 3.0 ^b^ | 4.0^a^ | 4.0 ^a^ | 4.0 ^a^ |
| 4 | 0 ^d^ | 1.0 ^cd^ | 1.33 ^bc^ | 0 ^d^ | 2.0 ^bc^ | 3.0 ^ab^ | 4.0 ^a^ | 4.0 ^a^ | 4.0 ^a^ |
