# Supplementary figures and images for: Infectious bursal disease virus: predicting viral pathotype using machine learning models focused on early changes in total blood cell counts
Source: Vet Res. 2023 Oct 30;54:101. doi: 10.1186/s13567-023-01222-5 (PMC10614337; doi:10.1186/s13567-023-01222-5)

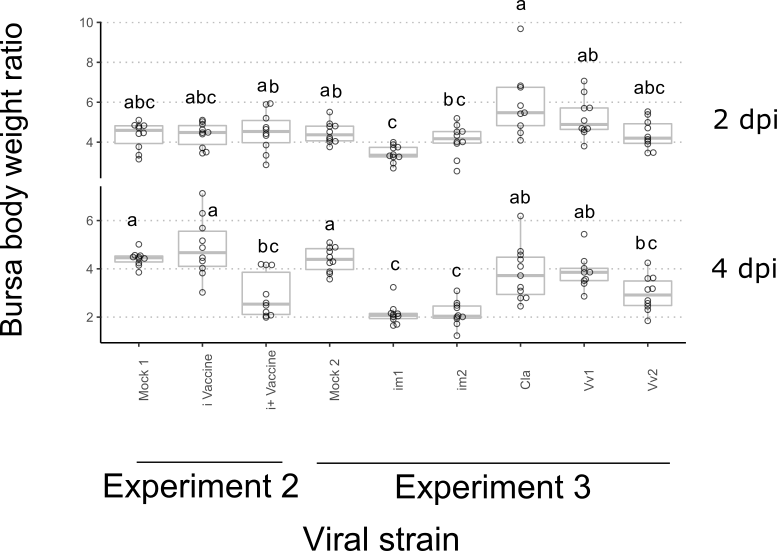

Supplement: Supplementary file 6 — Additional file 6: Bursa body weight ratio at 2 and 4 dpi (groups with at least one letter in common did not show any significant statistical variation of their median value). [file 13567_2023_1222_MOESM6_ESM.docx]

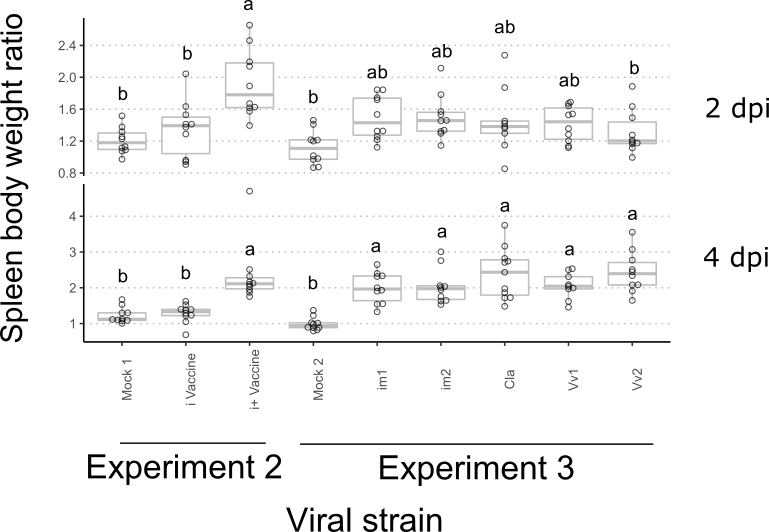

Supplement: Supplementary file 7 — Additional file 7: Spleen body weight ratio at 2 and 4 dpi (groups with at least one letter in common did not show any significant statistical variation of their median value). [file 13567_2023_1222_MOESM7_ESM.docx]

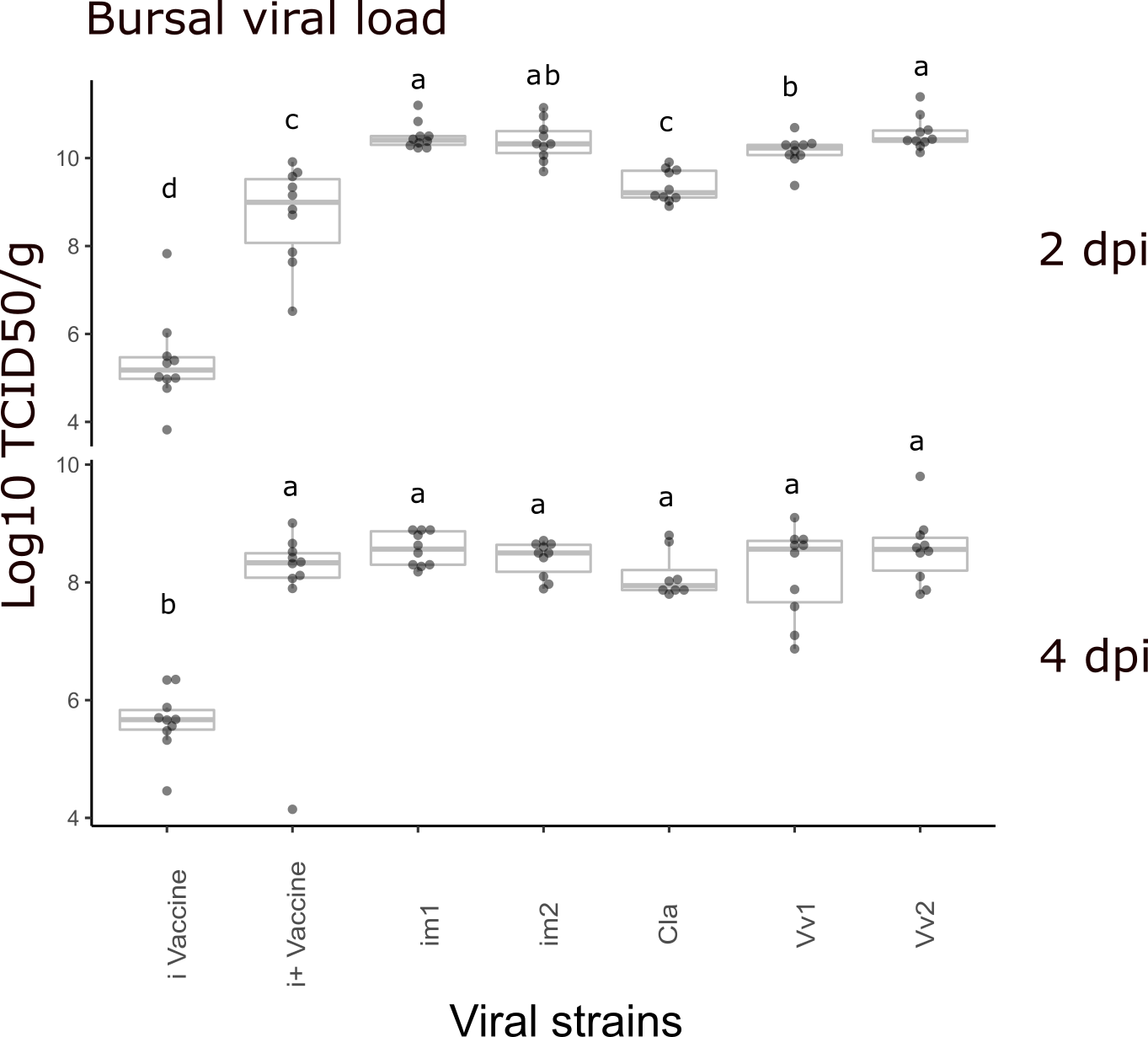

Supplement: Supplementary file 11 — Additional file 11: Bursal viral load at 2 and 4 dpi (experiment 2 and 3) (groups with at least one letter in common did not show any significant statistical variation of their median value). [file 13567_2023_1222_MOESM11_ESM.docx]

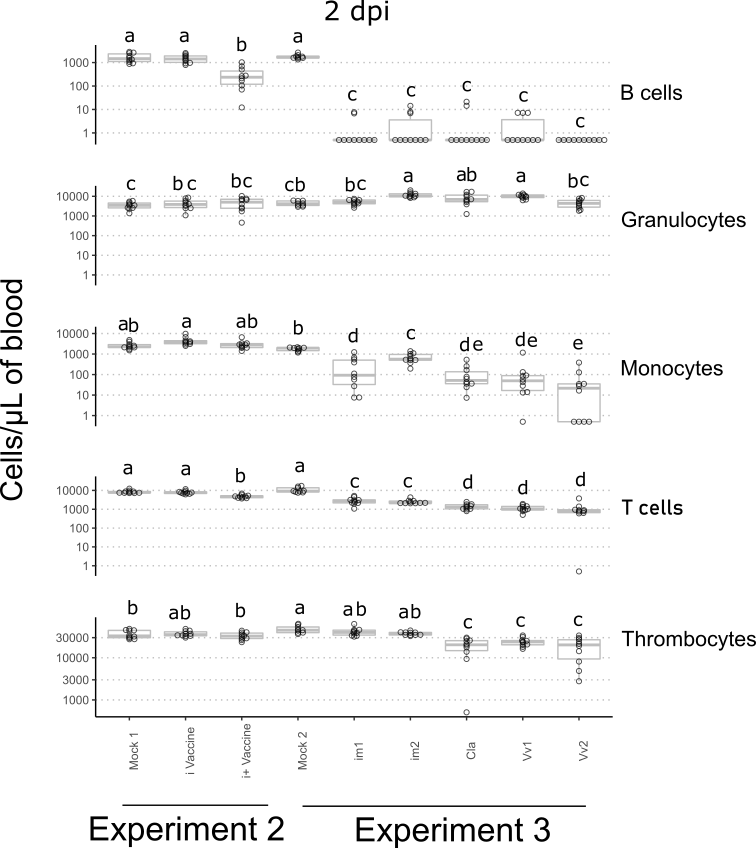

Supplement: Supplementary file 12 — Additional file 12: Blood cells concentration at 2 dpi (experiments 2 and 3) (groups with at least one letter in common did not show any significant statistical variation of their median value). [file 13567_2023_1222_MOESM12_ESM.docx]

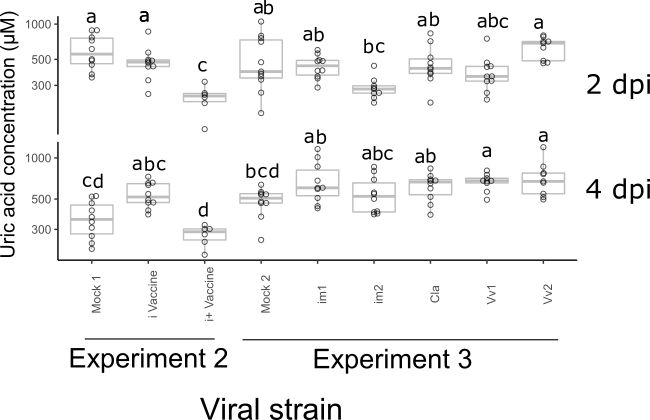

Supplement: Supplementary file 13 — Additional file 13: Uric acid blood concentration at 2 and 4 dpi (experiments 2 and 3) (groups with at least one letter in common did not show any significant statistical variation of their median value) [file 13567_2023_1222_MOESM13_ESM.docx]

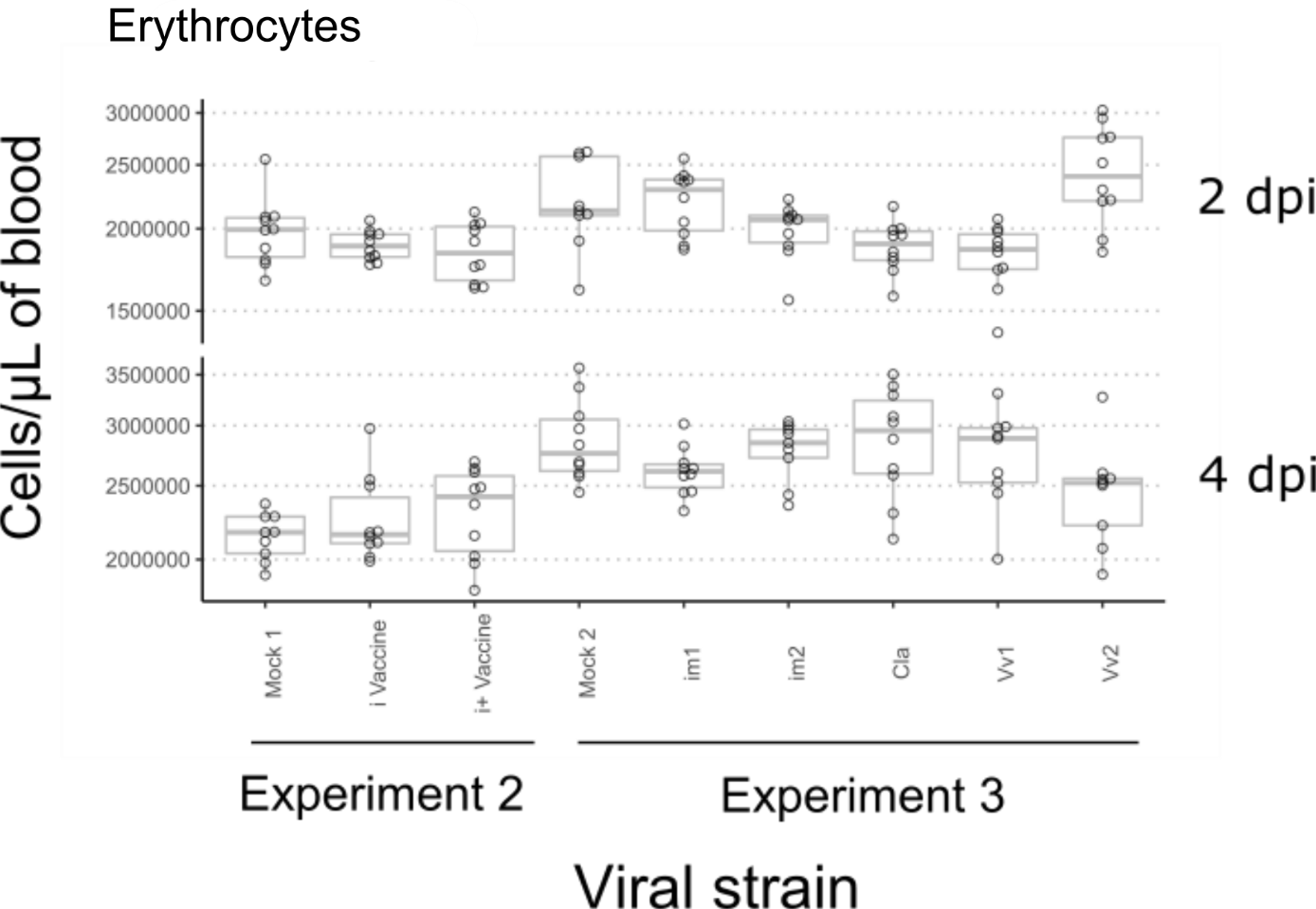

Supplement: Supplementary file 14 — Additional file 14: Erythrocytes blood concentration at 2 and 4 dpi (no statistically significant difference between the infected groups and the mocks was observed) (groups with at least one letter in common did not show any significant statistical variation of their median value). [file 13567_2023_1222_MOESM14_ESM.docx]
