# Supplementary material for: Infectious bursal disease virus: predicting viral pathotype using machine learning models focused on early changes in total blood cell counts
Source: Vet Res. 2023 Oct 30;54:101. doi: 10.1186/s13567-023-01222-5 (PMC10614337; doi:10.1186/s13567-023-01222-5)
Supplement: Supplementary file 8 — Additional file 8: Models performance at 2 days post-infection with the following parameters taken into account: bursal viral load, uricemia, blood cells concentrations (all), clinical score. Cla gathered the animals infected by the Cla strain, im those infected by im1 or im2 strains, i those infected by i vaccine strain, i+ those infected by i+ vaccine and Vv those infected by Vv1 or Vv2 strains. [file 13567_2023_1222_MOESM8_ESM.docx]

| Model | %.all | %.cla | %.i | %.i+ | %.im | %.vv |
| --- | --- | --- | --- | --- | --- | --- |
| naive_bayes | 85.3 | 75.9 | 90.7 | 89.5 | 95.3 | 81.5 |
| lda2 | 82.7 | 64.6 | 91.2 | 91.1 | 94.8 | 78.4 |
| kernelpls | 67.6 | 0.0 | 72.4 | 88.3 | 71.6 | 64.9 |
| rf | 89.7 | 91.7 | 86.7 | 93.9 | 94.3 | 90.6 |
| treebag | 87.4 | 89.1 | 84.8 | 82.5 | 94.2 | 91.2 |
| C5.0 | 85.1 | 92.4 | 85.5 | 70.8 | 92.3 | 90.4 |
| kknn | 79.8 | 47.9 | 93.5 | 89.2 | 94.3 | 71.9 |
| svmLinear | 83.6 | 73.1 | 92.6 | 81.7 | 95.9 | 81.0 |
| nnet | 84.0 | 72.1 | 91.1 | 91.2 | 94.9 | 79.8 |
| mlpML | 82.8 | 64.3 | 90.6 | 93.6 | 97.0 | 74.2 |
