# Supplementary material for: Infectious bursal disease virus: predicting viral pathotype using machine learning models focused on early changes in total blood cell counts
Source: Vet Res. 2023 Oct 30;54:101. doi: 10.1186/s13567-023-01222-5 (PMC10614337; doi:10.1186/s13567-023-01222-5)
Supplement: Supplementary file 9 — Additional file 9: Models performance at 4 days post-infection with the following parameters taken into account: bursal viral load, uricemia, blood cells concentrations (all), clinical score. Cla gathered the animals infected by the Cla strain, im those infected by im1 or im2 strains, i those infected by i vaccine strain, i+ those infected by i+ vaccine and Vv those infected by Vv1 or Vv2 strains. [file 13567_2023_1222_MOESM9_ESM.docx]

| Model | %.all | %.cla | %.i | %.i+ | %.im | %.vv |
| --- | --- | --- | --- | --- | --- | --- |
| naive_bayes | 82.7 | 65.3 | 97.5 | 97.2 | 82.1 | 81.0 |
| lda2 | 78.5 | 58.2 | 86.2 | 79.0 | 84.6 | 86.5 |
| kernelpls | 73.2 | 100.0 | 87.7 | 99.1 | 57.9 | 85.0 |
| rf | 84.5 | 82.0 | 95.9 | 98.0 | 86.2 | 78.7 |
| treebag | 78.4 | 58.2 | 97.8 | 88.8 | 87.6 | 74.5 |
| C5.0 | 77.8 | 54.1 | 92.0 | 84.5 | 88.6 | 75.6 |
| kknn | 79.0 | 60.9 | 88.5 | 92.1 | 82.9 | 80.5 |
| svmLinear | 80.0 | 66.8 | 86.7 | 83.2 | 84.2 | 86.8 |
| nnet | 82.5 | 73.5 | 86.4 | 88.2 | 88.6 | 84.7 |
| mlpML | 83.5 | 80.0 | 88.0 | 87.3 | 88.0 | 83.3 |
