# Supplementary material for: Infectious bursal disease virus: predicting viral pathotype using machine learning models focused on early changes in total blood cell counts
Source: Vet Res. 2023 Oct 30;54:101. doi: 10.1186/s13567-023-01222-5 (PMC10614337; doi:10.1186/s13567-023-01222-5)
Supplement: Supplementary file 10 — Additional file 10: Models performance at 4 days post-infection with the following parameters taken into account: uricemia, blood cells concentrations (t, b, g, tr). Cla gathered the animals infected by the Cla strain, im those infected by im1 or im2 strains, i those infected by i vaccine strain, i+ those infected by i+ vaccine and Vv those infected by Vv1 or Vv2 strains. [file 13567_2023_1222_MOESM10_ESM.docx]

| Model | %.all | %.cla | %.i | %.i+ | %.im | %.vv |
| --- | --- | --- | --- | --- | --- | --- |
| naive_bayes | 82.0 | 55.5 | 99.5 | 90.5 | 87.8 | 80.2 |
| lda2 | 79.3 | 52.2 | 98.8 | 85.4 | 76.4 | 89.7 |
| kernelpls | 73.7 | NaN | 97.4 | 90.7 | 57.7 | 80.7 |
| rf | 83.0 | 67.1 | 100.0 | 94.8 | 89.1 | 77.4 |
| treebag | 77.7 | 51.7 | 99.8 | 88.4 | 87.6 | 75.1 |
| C5.0 | 75.9 | 48.7 | 90.7 | 83.0 | 87.5 | 75.5 |
| kknn | 78.8 | 40.9 | 99.0 | 89.1 | 88.3 | 81.4 |
| svmLinear | 83.9 | 57.2 | 100.0 | 90.4 | 90.2 | 88.6 |
| nnet | 81.0 | 60.8 | 98.2 | 88.4 | 84.3 | 81.5 |
| mlpML | 79.4 | 62.0 | 89.0 | 83.4 | 83.9 | 77.7 |
